# Genetic replacement of surfactant protein-C reduces respiratory syncytial virus induced lung injury

**DOI:** 10.1186/1465-9921-14-19

**Published:** 2013-02-12

**Authors:** Stephan W Glasser, Albert P Senft, Melissa D Maxfield, Teah L Ruetschilling, John E Baatz, Kristen Page, Thomas R Korfhagen

**Affiliations:** 1 Cincinnati Children’s Hospital Medical Center, Perinatal Institute, Division of Neonatology, Perinatal and Pulmonary Biology, MLC7029, 3333 Burnet Avenue, Cincinnati, OH, 45229-3039, USA; 2 Lovelace Respiratory Research Institute, Albuquerque, NM, USA; 3 Medical University of South Carolina, Charleston, South Carolina, USA

**Keywords:** Surfactant protein-C, Respiratory syncytial virus, Type II cells, Lung inflammation, Interstitial lung disease, Clara cell secretory protein (CCSP)

## Abstract

**Background:**

Individuals with deficiencies of pulmonary surfactant protein C (SP-C) develop interstitial lung disease (ILD) that is exacerbated by viral infections including respiratory syncytial virus (RSV). SP-C gene targeted mice (*Sftpc -/-*) lack SP-C, develop an ILD-like disease and are susceptible to infection with RSV.

**Methods:**

In order to determine requirements for correction of RSV induced injury we have generated compound transgenic mice where SP-C expression can be induced on the *Sftpc -/-* background (SP-C/*Sftpc -/-*) by the administration of doxycycline (dox). The pattern of induced SP-C expression was determined by immunohistochemistry and processing by Western blot analysis. Tissue and cellular inflammation was measured following RSV infection and the RSV-induced cytokine response of isolated *Sftpc +/+* and *-/-* type II cells determined.

**Results:**

After 5 days of dox administration transgene SP-C mRNA expression was detected by RT-PCR in the lungs of two independent lines of bitransgenic SP-C/*Sftpc -/-* mice (lines 55.3 and 54.2). ProSP-C was expressed in the lung, and mature SP-C was detected by Western blot analysis of the lavage fluid from both lines of SP-C/*Sftpc -/-* mice. Induced SP-C expression was localized to alveolar type II cells by immunostaining with an antibody to proSP-C. Line 55.3 SP-C/*Sftpc -/-* mice were maintained on or off dox for 7 days and infected with 2.6x10^7^ RSV pfu. On day 3 post RSV infection total inflammatory cell counts were reduced in the lavage of dox treated 55.3 SP-C/*Sftpc -/-* mice (p = 0.004). The percentage of neutrophils was reduced (p = 0.05). The viral titers of lung homogenates from dox treated 55.3 SP-C/*Sftpc -/-* mice were decreased relative to 55.3 SP-C/*Sftpc -/-* mice without dox (p = 0.01). The cytokine response of *Sftpc -/-* type II cells to RSV was increased over that of *Sftpc +/+* cells.

**Conclusions:**

Transgenic restoration of SP-C reduced inflammation and improved viral clearance in the lungs of SP-C deficient mice. The loss of SP-C in alveolar type II cells compromises their response to infection. These findings show that the restoration of SP-C in *Sftpc -/-* mice in response to RSV infection is a useful model to determine parameters for therapeutic intervention.

## Introduction

SP-C is an abundant surfactant associated lipoprotein expressed in alveolar type II epithelial cells that synthesize and secrete pulmonary surfactant into the airspace. While SP-C confers dramatic surface activity it is not essential for perinatal transition to breathing. Individuals lacking SP-C survive but develop a variety of interstitial or fibrotic lung disease outcomes [1]. SP-C related disease can occur as acute postnatal respiratory distress or with slow onset that is identified as respiratory insufficiency during childhood through adulthood [2,3]. The molecular pathogenesis of SP-C related lung disease includes loss of gene expression without defined mutations, decreased expression from non-coding promoter mutations, and most commonly mutations in the SP-C coding gene (*SFTPC*) [4-6]. The *SFTPC* mutations have distinct consequences, altering mRNA splicing, proprotein structure, protein expression, or processing. These mutation-derived defects induce both a cellular stress injury due to altered structure and processing of the SP-C proprotein and a decrease in the amount of functional mature SP-C lipoprotein released into the airspace. SP-C deficient mice (*Sftpc -/-)* were generated in order to determine the role of SP-C in pulmonary homeostasis and distinguish injury based upon deficiency versus injury induced by aberrant SP-C expression products. On the 129S6 background *Sftpc -/-* mice develop parenchymal lung injury with age that is similar to the interstitial lung disease (ILD) reported for individuals with deficiencies of SP-C or *SFTPC* mutations [7,8].

Respiratory crises in affected *SFTPC* individuals have been linked to viral infections that include influenza, parainfluenza, and RSV [1,9,10]. RSV is a common pediatric respiratory pathogen that elicits an intense inflammatory injury primarily in infants but can also re-infect and complicate adult chronic obstructive pulmonary disease (COPD). RSV infection induces airway inflammation, wheezing, and in some cases inflammation involving the lower airspaces [11,12]. There is a growing appreciation of the role of the alveolar epithelium in the production of inflammatory mediators and the regulation of innate defense [13]. The infection related exacerbations of affected individuals indicate that SP-C confers protection and may limit infection from progressing to the alveolar compartment.

*Sftpc -/-* mice are susceptible to RSV infection with decreased viral clearance and a more robust and sustained pulmonary inflammation [14]. The host pulmonary immune response to RSV is complex and for both humans and mice includes pathogen receptor Toll-like receptor-3 (TLR3) activation of inflammatory gene expression and recruitment of the innate cellular defenses [14]. *Sftpc -/-* mice had increased lung inflammation when challenged with a synthetic ligand for TLR3, and SP-C phospholipid preparations blocked TLR3 mediated signaling *in vitro*. The increased severity of RSV infection in the *Sftpc -/-* mice and the impact of viral infection in *SFTPC* related patients indicate that SP-C is a significant component of the pulmonary innate immune system.

We hypothesized that genetic restoration of SP-C would reduce RSV injury in the lungs of SP-C deficient mice. To test that hypothesis transgenic mice were generated that conditionally express and process proSP-C in the lungs of SP-C deficient mice. The data show that SP-C expression for seven days was sufficient to provide protection from RSV induced injury. Development of this *in vivo* model to genetically regulate SP-C expression will aid in designing therapeutic intervention and identifying RSV-induced genes regulated by SP-C.

## Methods

### Inducible SP-C transgene construction and genetic crosses to establish a regulated SP-C model

CCSP-rtTA transgenic mice that express the tetracycline transactivator protein were previously generated and characterized. The CCSP-rtTA transgenic line expresses the tetracycline transactivator within twenty-four hours of administration of the tetracycline analog-doxycycline (dox) primarily in the alveolar epithelium rather than bronchiolar epithelial expression seen for the endogenous CCSP gene [15,16]. An available SP-C-rtTA transgenic line expressed low levels of the rtTA mRNA without dox induction and thus due to the background leak, was less useful for the studies in this manuscript [17]. Because the CCSP-rtTA transgenic line was tightly regulated by dox administration and the transgene was also expressed in the SP-C producing type II cells the CCSP-rtTA transactivator line was used to breed and establish the model of inducible SP-C replacement. A second transgene was constructed that would conditionally express SP-C in response to the CCSP-rtTA activator. The full-length murine SP-C cDNA was cloned directionally into unique EcoR1 (5’) and XbaI (3’) restriction sites downstream of the tetO_7_ CMV promoter in the plasmid pUHD15-1 [15,18]. Transcriptional activation to express the linked SP-C cDNA requires binding of the rtTA protein in the presence of dox to the seven binding sites (tetO_7_) adjacent to the CMV promoter. XhoI-HindIII double digestion was used to release the tetO_7_CMV-SP-C transgene from the bacterial plasmid backbone for purification and microinjection. Potential transgene founder animals were screened by PCR analysis of tail clip DNA. Transgene positive tetO_7_CMV-SP-C founder animals were bred to *Sftpc -/-* mice that were then crossed to the CCSP-rtTA/*Sftpc -/-* mice in order to generate compound genetically modified mice carrying both transgenes and the targeted *Sftpc -/-* locus. These CCSP-rtTA/tetO_7_-SP-C/*Sftpc -/-* mice were used to test for doxycycline (dox) induction of transgene mediated SP-C expression. Animal studies were performed under protocols approved by the Children’s Hospital Research Foundation Institutional Animal Care and Use Committee (Cincinnati, Ohio).

### Detection of inducible transgene mRNA and protein expression

The CCSP-rtTA/tetO_7_-SP-C/*Sftpc -/-* mice were placed on commercial dox supplemented chow for five days prior to analysis. SP-C mRNA expression was assessed by RTPCR of cDNA prepared from total lung RNA isolated from *Sftpc +/+*, *Sftpc -/-*, single transgene *Sftpc -/-* and compound CCSP-rtTA/tetO_7_-SP-C/*Sftpc -/-* mice fed either normal or dox chow. Expression and processing of the proSP-C protein to the mature SP-C was assessed by Western blot. An antibody to the 21kD proSP-C (Seven Hills Bioreagents, WRAB-9337) was used to identify expression of the full-length proSP-C in lung tissue and a second antibody (Seven Hills Bioreagents, WRAB-76694) to detect the 4.5kD processed mature SP-C in both tissue and bronchoalveolar lavage fluid (BALF).

### Immunohistochemistry and localization of induced SP-C protein in the lungs

The lungs of line 55.3 CCSP-rtTA/tetO_7_-SP-C/*Sftpc -/-* and *Sftpc +/+* mice on or off of dox were inflation fixed, paraffin embedded and five micron sections processed for immunohistochemistry with the anti proSP-C antibody. The primary antibody - WRAB-9337 was used at 1:1,000 dilution overnight at four degrees and the secondary HRP conjugated goat anti-rabbit antibody used at 1:200 dilution for thirty minutes and immunoreactivity visualized by reaction with nickel enhanced diaminobenzidine (Sigma-Aldrich Co.) and nuclear fast red counter stain. Lung histopathology after infection was visualized using conventional hematoxylin and eosin staining. Airway goblet cell transformation was determined by alcian blue histochemical staining (Poly Scientific R&D Corp.).

### RSV infection, viral titer, and inflammation of CCSPrtTA/tetO_7_-SP-C/*Sftpc -/-* mice

Seven-week old mice were placed on dox chow for seven days, anesthetized by isoflurane inhalation and then infected by oral aspiration with 2.6 × 10^7^ pfu or RSV cell growth media as non-viral control [14]. Three days after RSV infection viral titers were quantified by serial dilution of lung homogenates in triplicate on Hep2 cells. Plaques were visualized by immuno-staining of fixed cells with a monoclonal antibody to RSV F protein (Fitzgerald Industries Int. Inc., 10R-R113B) and counterstained with nuclear fast red (n = 5 mice per group) [14].

Bronchoalveolar lavage fluid (BALF) was obtained with three sequential lavages using one ml aliquots of phosphate buffered saline (3 instillation cycles with each aliquot). Total cell counts were determined by visual counting using a hemocytometer and differential cell counts determined from cytospin preparations visualized with DiffQuik staining (Diff Quick Stain Set, Siemens).

### Preparation of SP-C-phospholipid liposomes and inhibition of RSV infection

Native SP-C was purified by C8 liquid chromatography of bovine lung lavage, as previously described [14]. Briefly, dipalmitoylated SP-C was identified as a single peak at m/z 4058.4 Da by matrix assisted laser desorption ionization time of flight (MALDI-TOF) mass spectrometry. The purified SP-C stock solution was free of detectable levels of endotoxin. An SP-C stock solution in 7:1:1 methanol/chloroform/water (v/v/v) was added to 10 mg/ml stock solutions of phospholipid liposomes prepared with representative surfactant phospholipids, dipalmitoyl-phosphatidylcholine (DPPC) and palmitoyloleoyl-phosphatidylcholine (POPC) (Avanti Polar Lipids) at a DPPC:POPC molar ratio of 7:3 with isolated SP-C incorporated 5% by weight. DPPC/POPC liposomes (7:3 mol/mol) without SP-C were used as control liposomes. Liposome preparations were dried under nitrogen at 45°C. Endotoxin free Dulbecco’s phosphate buffered saline was added to the dried film at 45°C, vortexed for 10s and sonicated for 10s to yield vesicle preparations at a final concentration of 25 mg/ml phospholipid. The effect of SP-C on inhibition of viral infection *in vitro* was determined. 60,000 Hep2 cells were seeded per well of 12 well dish. The next day synthetic phospholipid liposomes with or without SP-C (25ug/ml) were overlaid onto cell monolayers and incubated for one hour prior to RSV infection at a multiplicity of infection (MOI) of 2, held at 37°, 5% CO_2_ for two days and the monolayers fixed, stained with the anti-RSV F protein antibody, counterstained and plaques counted. Experiments were performed in triplicate.

### Determination of cytokine expression by isolated type II cells of *Sftpc +/+ and -/-* mice following infection

Type II cells were isolated using minor modifications of a published procedure [19,20]. Following humane euthanasia, the instillation of Dispase (Gibco) into the lungs of five-week old mice was used to initiate alveolar enzymatic digestion of epithelial cells followed by instillation of low melt agarose to block release and contamination from airway respiratory epithelial cells. Digested cell suspensions were filtered, overlaid onto to CD45, CD32 antibody coated plates to capture non-epithelial cells. The non-adherent type II cells were recovered by centrifugation and resuspended in BEGM culture media (Lonza). The type II cells were seeded (150,000 cells per well) onto forty-eight well dishes coated with a 70:30 vol/vol mixture of Cultrex (Trevigen Inc.): rat-tail collagen (BD Bioscience) basement membrane matrix. Cells were maintained for three days to allow recovery of the differentiated type II cell phenotype [19]. Triplicate wells for both *Sftpc* +/+ and -/- cells were subject to infection at a MOI of 2. The media was removed at 24 hours post infection. Cell debris was removed by centrifugation and cytokine concentrations in the supernatant determined by ELISA assay (R&D systems).

### Statistical analysis

Potential statistical significance was determined using one-way ANOVA or Student’s t test.

P values at or below 0.05 were accepted as significant.

## Results

### Generation of inducible SP-C replacement model: identification of founder double transgene and *Sftpc-/-* compound mice

The CCSP-rtTA transgenic line that expresses the tetracycline transactivator protein in distal airway and alveolar epithelial cells was previously generated and shown to primarily direct alveolar specific expression of rtTA rather than the bronchiolar-Clara cell expression for the endogenous CCSP gene [15,16]. Thus the CCSP-rtTA transgenic mice would potentially activate expression of the SP-C responder transgene (tetO_7_CMV-SP-C) in the desired surfactant producing type II cells.

Seven tetO_7_CMV-SP-C transgene founder mice were identified. Four of the founder tetO_7_CMV-SP-C mice were successfully bred to *Sftpc -/-* mice on the 129S6 background and then crossed with CCSP-rtTA/*Sftpc -/-* mice in order to test F1 offspring for inducible transgene SP-C expression to selectively replace SP-C in the lungs of the SP-C deficient, *Sftpc -/-* mice.

**Figure 1:**
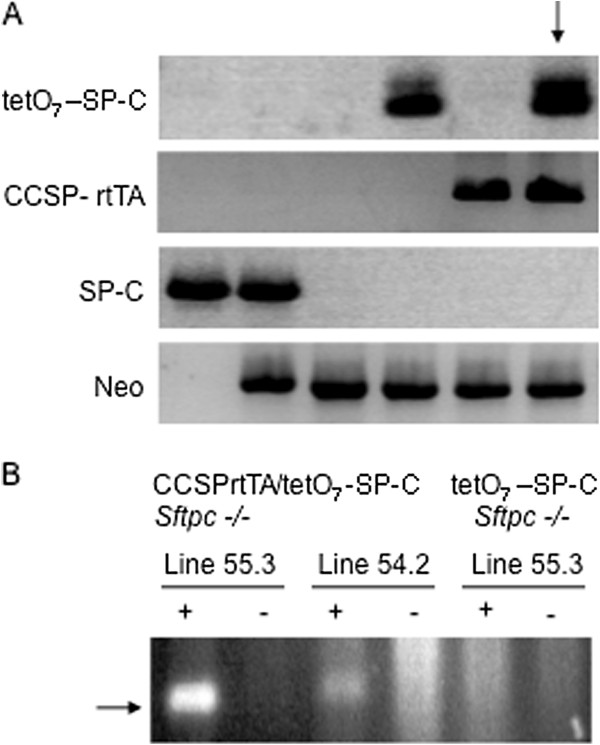
**Identification of transgenic mice that express SP-C in response to doxycycline administration.** Panel **A**: PCR results identify founder transgenic *Sftpc -/-* mice carrying either the dox responsive SP-C cDNA (tetO_7_-SP-C), the CCSP promoter directing expression of the tetracycline transactivator (CCSP-rtTA), or a mouse positive for both transgenes and the disrupted *Sftpc* locus (neomycin positive, SP-C negative) indicated by the arrow. Panel **B**: Lung cDNA from transgenic CCSP-rtTA/tetO_7_-SP-C -*Sftpc -/-* mice was assayed for dox induced expression of SP-C. Two CCSP-rtTA/tetO_7_-SP-C -*Sftpc -/-* founder lines, 55.3 and 54.2 expressed SP-C when fed dox chow (+ lanes). No SP-C expression was detected in the absence of dox (-lanes), or in the lungs of single transgenic tetO_7_-SP-C-*Sftpc -/-* mice on or off dox (last two lanes). Arrow indicates SP-C cDNA band.

SP-C negative and neomycin positive result identifies the disrupted *Sftpc* locus (Figure 1A, lane 3). As depicted in Figure 1A, single transgene tetO_7_CMV-SP-C mice on *Sftpc -/-* background were identified in lane 4 and single transgene CCSP-rtTA mice on *Sftpc -/-* background, lane 5. Arrow indicates desired genotype of double transgene positive and *Sftpc -/-* compound mice.

### mRNA expression

The CCSP-rtTA transactivator line used in this study is tightly regulated by dox administration, with expression of target tetO_7_-reporter transgene achieved within 18 hours of administration [17]. The F1 offspring of each line of CCSP-rtTA/tetO_7_-SP-C/*Sftpc -/-* mice were provided commercial dox supplemented chow for five days and inducible SP-C expression tested by RTPCR analysis of total lung cDNA. Two founder CCSP-rtTA/tetO_7_-SP-C lines, 54.2 and 55.3 expressed SP-C mRNA (lane 1 vs lane 3, Figure 1B). No SP-C mRNA was detected in the lungs of either the line 55.3/*Sftpc -/-* or 54.2/*Sftpc -/-* mice without dox (lanes 2 and 4, Figure 1B) or in single transgene tetO_7_CMV-SP-C/*Sftpc -/-* mice maintained on dox (lanes 5 and 6, Figure 1B). Thus re-expression of SP-C is tightly controlled, requiring the presence of both transgenes and dox administration.

Both the 55.3/*Sftpc -/-* and 54.2/*Sftpc -/-* lines were bred onto the 129S6/*Sftpc -/-* background for 9 generations to generate genetically uniform lines of CCSP-rtTA/tetO_7_-SP-C/*Sftpc -/-* mice. The backcrosses established the inducible transgenes on the susceptible 129S6 strain of *Sftpc -/-* mice that develop ILD like disease with age [8].

### Protein expression

**Figure 2:**
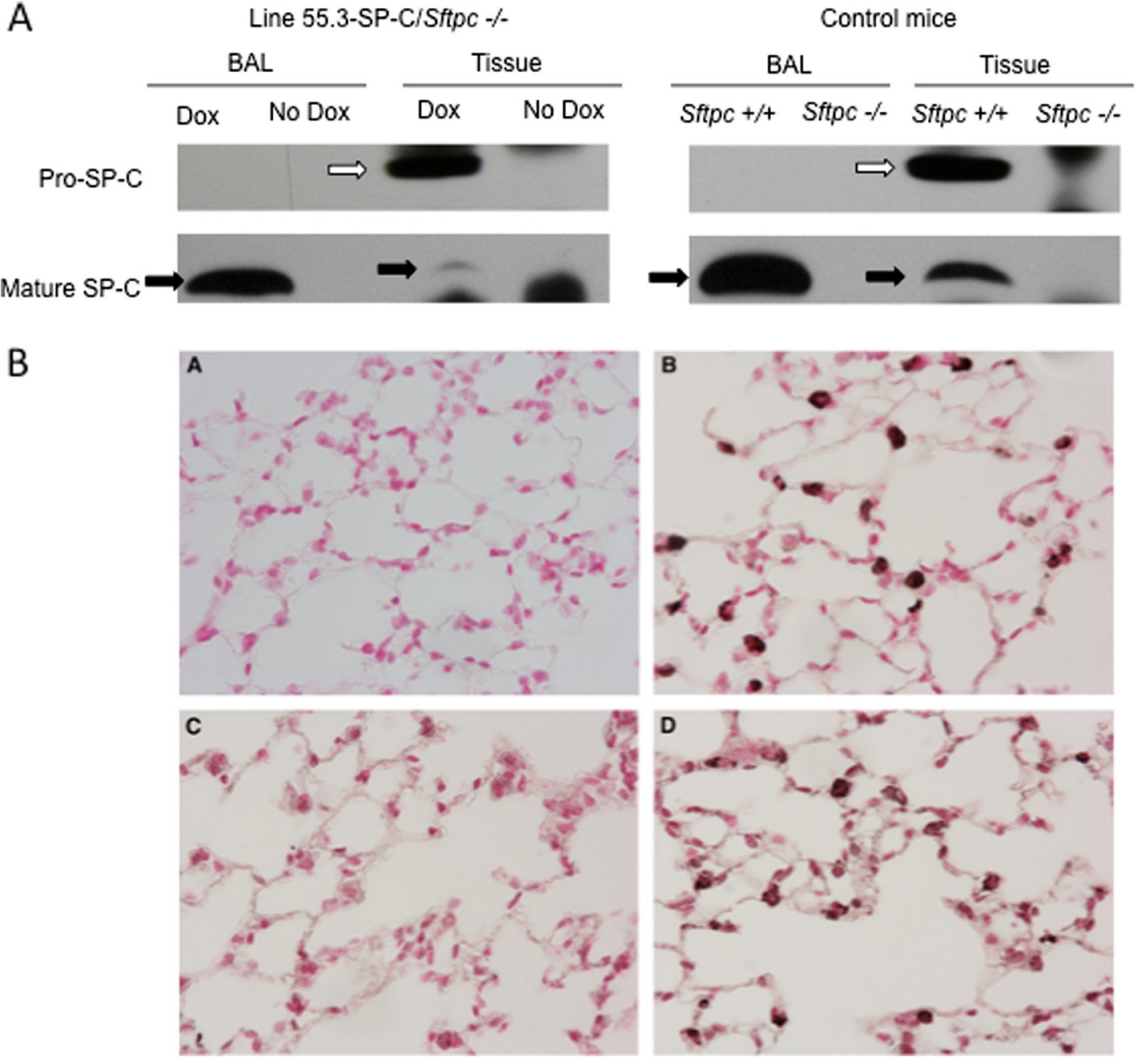
**Lung expression, processing, and localization of inducible SP-C expression.** Part **A**: Top row; Western blot analysis of proSP-C and processed SP-C expression in 55.3/*Sftpc -/-* mice. An antibody to proSP-C detected proSP-C in the lung homogenates from line 55.3/*Sftpc -/-* mice maintained on dox (white arrows). Lower row; Antibody to the proteolytically processed SP-C detected the mature 4.5kD SP-C in BAL fluid indicating secretion into the airspace (dark arrows). SP-C protein was only detected in the dox-treated 55.3/*Sftpc -/-* animals. Lung tissue and BAL fluid from *Sftpc +/+* mice are shown as positive controls and *Sftpc -/-* mice as negative controls for the specificity of SP-C immunostaining. Part **B**: Immuno-localization of dox-induced proSP-C in the lungs of line 55.3/*Sftpc -/-* mice: Panel **A**, *Sftpc +/+* lung with no primary antibody. Panel **B**, Lung tissue from *Sftpc +/+* mice ,Immunostaining with anti proSP-C antibody. Staining localized to alveolar type II cells. Panel **C**, No proSP-C staining was detected in the lungs of line 55.3/*Sftpc -/-* mice without dox induction. Panel **D**, proSP-C was detected in a type II cell pattern in lungs of dox treated line 55.3/*Sftpc -/-* mice, similar to *Sftpc +/+* mice in panel **B**. No alteration of lung morphology was observed by dox treatment.

SP-C is expressed as a 21kD proprotein that is proteolytically processed to a 4.5kD peptide that is incorporated into lamellar bodies for secretion. Using an antibody specific to proSP-C the 21kD proSP-C protein was detected by Western blot analysis of lung homogenates from the line 55.3 and line 54.2 SP-C/*Sftpc -/-* mice. Expression of proSP-C in line 55.3/*Sftpc -/-* mice is shown in the top panel of Figure 2A. Western blot analysis with an antibody specific to the mature 4.5kD form of SP-C detected significant SP-C in the BALF and a small amount of mature SP-C in lung tissue of the line 55.3/*Sftpc -/-* mice (lower panel, Figure 2A). These results indicate that the proSP-C is correctly expressed, processed to the mature protein in the lungs and secreted into the airspace. No proSP-C or mature SP-C protein was detected in the lungs of non-induced line 55.3/*Sftpc -/-* mice (no dox lanes). Antibody specificity was verified using lung tissue from *Sftpc +/+* mice as a positive control for detection of proSP-C in lung tissue and secreted mature SP-C in lavage fluid (right panel, Figure 2A). *Sftpc -/-* mice served as a negative control (right panel). Because induced SP-C expression appeared more robust in the line 55.3 /*Sftpc -/-* mice, this line was expanded and used in the following studies.

### *In situ* expression of the inducible SP-C transgene

Immunohistochemical staining was used in order to determine the level and pattern of SP-C expression induced in the lungs of line 55.3/*Sftpc -/-* mice after seven days of dox activation. Staining with an anti proSP-C antibody identified alveolar cell specific expression of SP-C (Figure 2B). The pattern of proSP-C staining for dox treated 55.3/*Sftpc -/-* mice (Figure 2B, panel D) was similar to the intensity and number of SP-C positive type II cells seen in wild type - *Sftpc +/+* mice (Figure 2B, panel B). No SP-C immunostaining was detected in the lungs of 55.3/*Sftpc -/-* mice without dox induction (Figure 2B, panel C). Sections treated without primary antibody, Figure 2B, panel A) were devoid of staining. In the current study induced SP-C was detected only in the lungs of the double transgenic line 55.3/*Sftpc -/-* mice administered dox indicating tight regulation of the proSP-C transgene. Dox treatment did not appear to alter lung morphology relative to *Sftpc +/+* mice (compare Figure 2B, panels C and D vs panel B).

### Effect of SP-C reconstitution on RSV mediated lung injury

**Figure 3:**
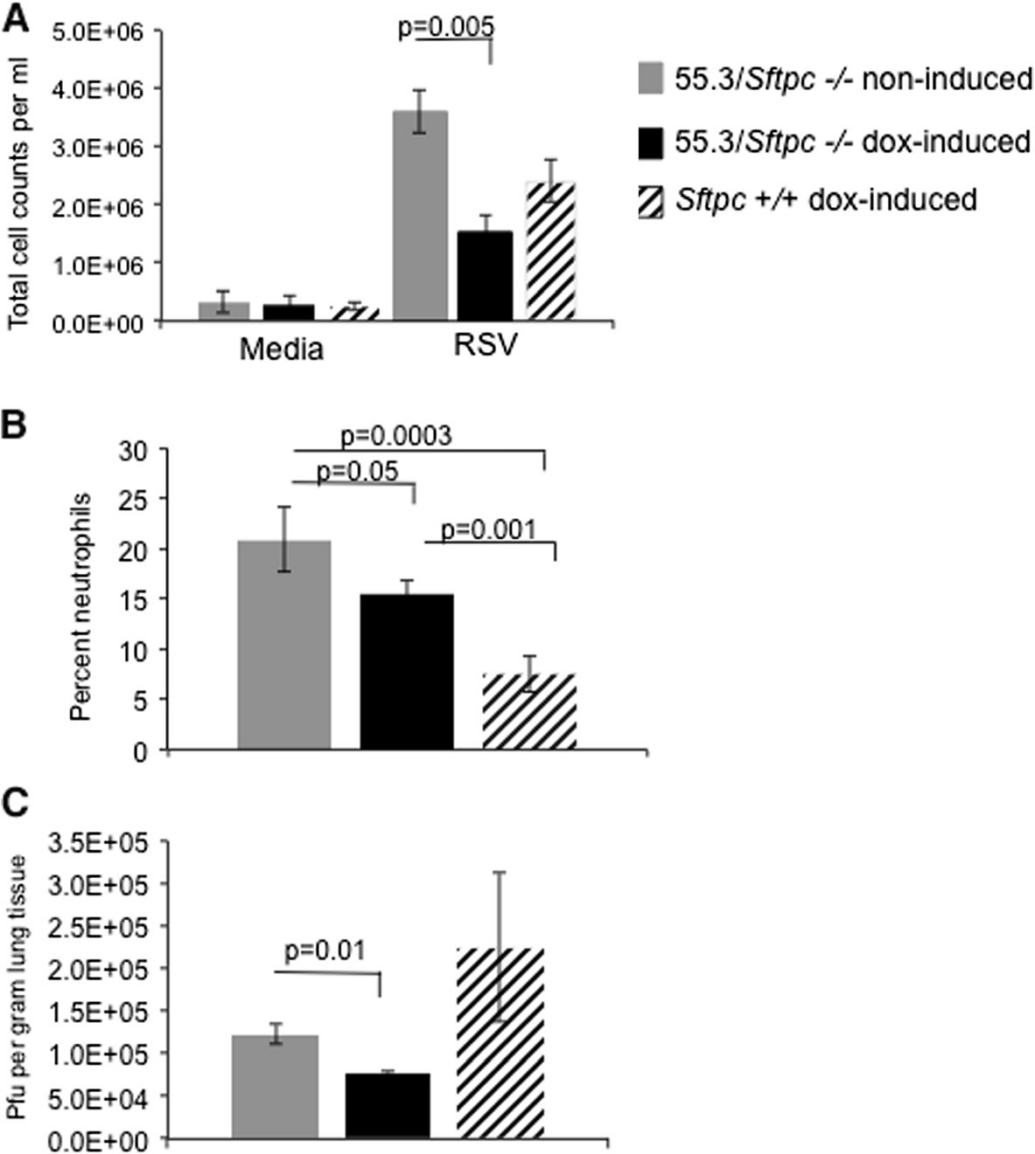
**Re-expression of SP-C reduces cellular inflammation and viral load in lungs of infected line 55.3/*Sftpc -/-*mice.** Following RSV infection, inflammatory cells in recovered BALF were analyzed for total cells (panel **A**) and the numbers of neutrophils determined from cytospins (panel **B**). Viral titers in infected lungs were determined by plaque assay of lung homogenates on day 3 post infection (panel **C**). Total cell counts from the BALF of media treated control mice were similar to *Sftpc +/+* mice on dox (lanes 1-3 panel **A**). Total cell counts and the percent neutrophils were increased in the BALF from RSV infected line 55.3/*Sftpc -/-* mice compared to *Sftpc +/+* mice on dox. Total cells, neutrophils and viral titer were all reduced in lungs of line 55.3/*Sftpc -/-* mice maintained on dox versus animals without dox (no SP-C) (5 mice per group).

Line 55.3/*Sftpc -/-* mice were maintained on continuous dox administration for seven days prior to RSV infection. Three days after infection the lungs were subject to bronchoalveolar lavage, or fixed for histology, or removed and homogenized for viral titer determination by *in vitro* RSV focus forming plaque assay. Cellular inflammation was reduced in the lungs of RSV infected line 55.3/*Sftpc -/-* mice maintained with dox. Total cell counts in the BALF of the dox treated line 55.3/*Sftpc -/-* mice were decreased by 58% and the percentage of neutrophils in recovered BALF was also decreased relative to non-dox treated line 55.3/*Sftpc -/-* mice (Figure 3). Viral loads were decreased by 40% in the lungs of dox activated line 55.3/*Sftpc -/-* mice relative to compound transgenic mice without dox activation (Figure 3, lower panel). Administration of dox to activate reporter gene expression was shown to not alter RSV infectivity [21]. Dox administration to line 55.3/*Sftpc -/-* and *Sftpc +/+* media control mice did not increase BALF inflammatory cell counts (Figure 3 top panel).

**Figure 4:**
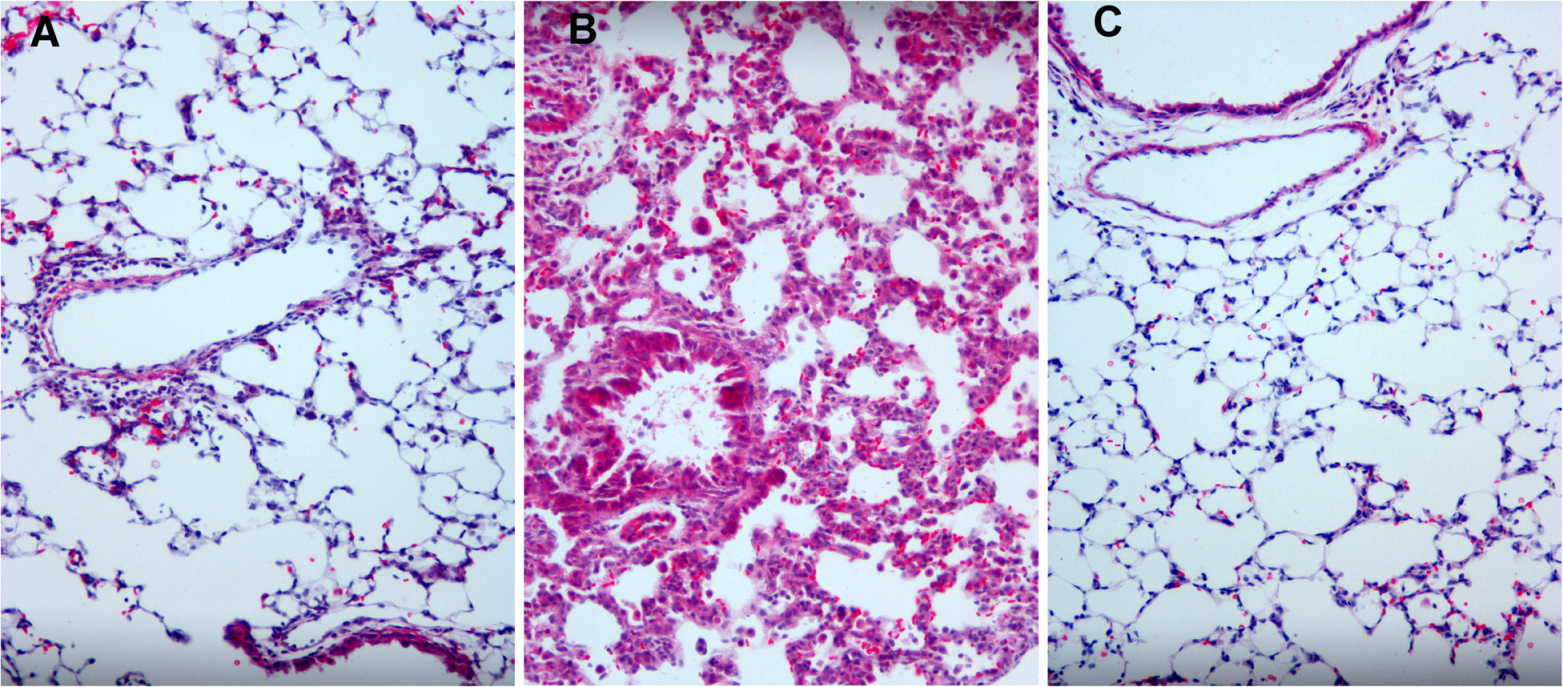
**Restoration of SP-C reduces RSV induced lung histopathology.** RSV induced mild perivascular, bronchiolar inflammatory cell infiltration in the lungs of *Sftpc +/+* mice (panel **A**). Lungs of the line 55.3/*Sftpc -/-* mice (no SP-C) without dox had interstitial inflammation and a diffuse cellular infiltration throughout the airspaces (panel **B**) similar to the previously reported histopathology in *Sftpc -/-* mice [14]. The lungs of 55.3/*Sftpc -/-* mice with dox-induced re-expression of SP-C prior to RSV infection had reduced observable histopathology (panel **C**). Presented sections are representative of sections from five mice.

Mild perivascular, peribronchiolar infiltrates were observed in the lungs of infected *Sftpc +/+* mice. In contrast, diffuse alveolar inflammatory infiltrates and interstitial inflammation was observed in the lungs of the line 55.3/*Sftpc -/-* mice without dox. The apparent inflammatory injury was decreased in the lungs of line 55.3/*Sftpc -/-* mice maintained on dox prior to RSV infection (Figure 4). The observed pathology in non-dox line 55.3/*Sftpc -/-* mice was similar to the increased injury originally reported for RSV infection of *Sftpc -/-* mice [14]. The reduction in observable tissue inflammation is consistent with the reduced viral titer and inflammatory cell counts with dox-induced restoration of SP-C.

### Isolated SP-C reduces infectivity *in vitro*

**Figure 5:**
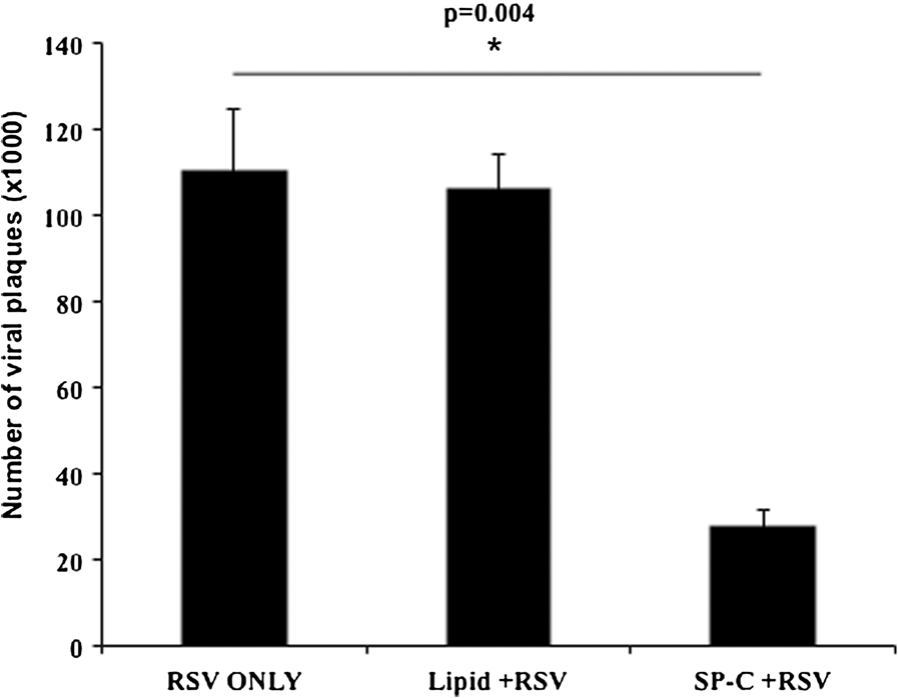
**SP-C inhibits viral infection in vitro.** Hep2 cells were infected with RSV alone, or with RSV following a one hour pre-treatment with phospholipid liposomes +/- SP-C. The synthetic phospholipid liposomes did not reduce viral plaque numbers while the liposome with incorporated SP-C reduced the viral plaque counts. N = 3

In order to determine if SP-C directly inhibits initial viral infection, the RSV permissive cell line Hep2 was pretreated with phospholipid liposomes or phospholipid liposomes with exogenous SP-C for one hour prior to viral infection (Figure 5). Exposure of the Hep2 cells to liposomes without SP-C did not alter the viral titer compared to RSV infection alone. However, liposomes with SP-C reduced the viral titer by 73%. The SP-C dependent reduction indicates that SP-C associated with surfactant phospholipids inhibits viral-epithelial cell interactions that are essential for infection.

### The type II cells from SP-C deficient mice have an increased inflammatory response to RSV

**Figure 6:**
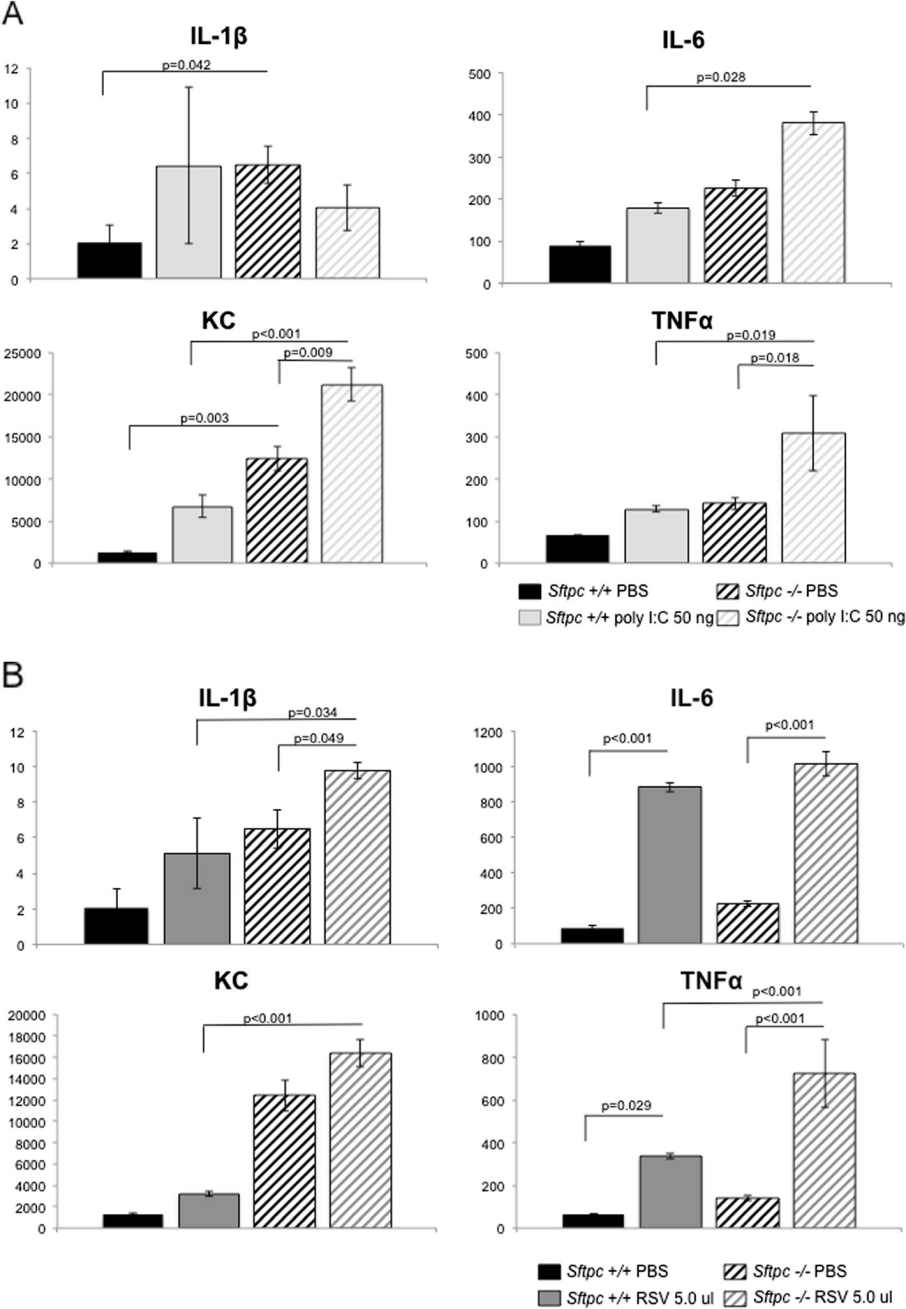
**SP-C deficiency increases the type II cell cytokine response to RSV infection and TLR3 challenge.** Cytokine levels were measured in the culture media of *Sftpc +/+* and *-/-* type II cells challenged with the TLR3 ligand poly I:C (Panel **A**) or by RSV infection (Panel **B**). Basal levels of cytokine expression were elevated in the media from *Sftpc -/-* type II cells and the response to poly I:C or virus was increased relative to the *Sftpc +/+* cells. Cytokine concentrations are in pg/ml (x axis). N = 3.

Isolated *Sftpc -/-* type II cells in culture had elevated intrinsic expression of cytokines IL-6, TNFα, KC and to a lesser extent IL1-β than type II cells from the lungs of *Sftpc +/+* mice (Figure 6). Upon challenge with the viral dsRNA surrogate poly (I:C) or RSV infection (Figure 6A and B respectively) the cytokine response by *Sftpc -/-* type II cells was increased relative to *Sftpc +/+* cells. These results indicate that the loss of SP-C renders the alveolar type II cell more susceptible to infection driven inflammation that occurs in part by TLR3 dependent activity (poly I:C ligand for TLR3).

## Discussion

SP-C deficient mice have increased inflammation and prolonged recovery from injury following infection with RSV [14]. A genetic model of regulated SP-C restoration was generated wherein proSP-C was expressed, processed to mature SP-C, and secreted in the airspace. When SP-C expression was induced in the lungs of *Sftpc -/-* mice prior to RSV infection, the pulmonary infiltration, viral titers, and morphologic indicators of injury were reduced. The increased susceptibility of *Sftpc -/-* mice to RSV and partial rescue by short-term alveolar expression supports the concept that SP-C contributes to innate alveolar defense against viral infection.

### Reduced expression of SP-C can compromise lung defense by multiple mechanisms

Pulmonary surfactant is essential for normal respiration and for protection from inspired microorganisms. Rare forms of familial ILD have been linked to decreased SP-C expression and indicate that SP-C is required for lung homeostasis. The age of onset and severity of disease can vary significantly between affected family members ranging from an immediate neonatal RDS to childhood or adult onset ILD/IPF [2]. This variable progression suggests the activity of additional disease modifiers.

The complex underlying molecular pathology of SP-C related ILD results from decreased *SFTPC* gene expression through mutations that alter the structure and abundance of either mRNA or protein. In addition, familial SP-C deficient ILD has been reported without any structural *SFTPC* mutation [4,5]. The affected individuals appear to be a true null phenotype. A recent report identifies individuals with SP-C deficiency due to clustered mutations in a distal upstream promoter region and implicates this region as an essential regulatory element for *SFTPC* gene expression [6]. Thus, the simple absence of SP-C can produce neonatal and/or childhood ILD.

The majority of individuals with SP-C related disease have mutations in the *SFTPC* gene. Mutations are primarily dispersed throughout the region downstream of the mature peptide altering mRNA splicing, proprotein processing, and proprotein structure and folding [3,22,23]. Mutations that alter processing or structure can induce both a cellular stress response as well as produce a net decrease in the amount of the secretory form of the protein. These distinct genetic alterations and resulting protein abnormalities may account for the reported pleiotropic phenotype. The *Sftpc -/-* mice have been used to explore the impact of the deficiency separate from deleterious effects of aberrant forms of proSP-C.

### SP-C abnormalities increase the severity of RSV lung disease

Viral infections are a risk factor for preexisting conditions that include immunosuppression, cystic fibrosis, or prematurity [11]. In a similar pattern, the pulmonary disease in young *SFTPC* patients can be complicated by influenza A/B virus or RSV infection. RSV infection is a common cause of respiratory infections in children that can vary from mild upper airway disease to severe bronchiolitis that requires hospitalization. RSV bronchiolitis during infancy also increases the incidence of chronic respiratory conditions such as wheezing, asthma, and allergy later into childhood [11,24]. RSV related exacerbation has occurred in affected individuals either with or without a defined *SFTPC* mutation [2,9,10]. In a separate study, expression of an *SFTPC* mutation *in vitro* rendered the cells more susceptible to RSV infection [25]. In support of the human clinical findings, a sub-strain of cattle with hereditary SP-C deficiency has an increased incidence of newborn respiratory distress. These cattle had increased fatal lung disease following an attempt to vaccinate against bovine RSV [26]. The findings from human case histories, as well as bovine and mouse model of SP-C deficiency indicate that SP-C is an important factor in both the control of RSV infection and recovery from RSV infection.

In the current study *Sftpc -/-* type II cells had increased basal levels of key cytokines and expressed increased cytokine levels when challenged with either synthetic dsRNA or upon RSV infection *in vitro*. Thus the absence of SP-C is sufficient to predispose the type II cell to aberrant inflammation upon viral infection. These findings indicate that type II cell function can contribute to alveolar defense during severe RSV infection that in part relies upon the immune-protective activity of SP-C.

### Restoration of SP-C in type II cells improves the outcome of RSV infection

RSV infection of *Sftpc +/+* and *-/-* mice produced increased tissue and cellular inflammation in the lungs of the *Sftpc -/-* mice [14]. Recovery from RSV related disease was incomplete in the lungs of *Sftpc -/-* mice thirty days after initial infection. As a first step to determine how SP-C confers alveolar protection, we have transiently replaced SP-C in a cell correct manner *in vivo* to assess reduction of inflammation and viral clearance. The SP-C proprotein was expressed and processed to the mature peptide that was secreted and detected in the BALF. SP-C expression was only detected following dox administration consistent with tight regulation of the SP-C transgene. Tissue inflammation and inflammatory cell infiltration was decreased on day 3 post-infection in the line 55.3/*Sftpc -/-* mice on doxycycline while more severe histopathology was apparent in the same mice without the induced SP-C expression. The incomplete reversal of RSV-induced injury may reflect the pattern of induced transgenic SP-C expression in a subset of type II cells when compared to wild type mice. The presence of the CCSP-rtTA transgene has been reported to alter alveolar size in mice on a different genetic background than the mice in this study and by inbreeding of lines of mice [27]. Based upon histological examination of three sections from three representative mice in each group there were no indications of differences in morphology between either the CCSP-rtTA or tetO_7_-SP-C transgenic mice or in two independent lines of the double transgenic-*Sftpc -/-* mice (lines 55.3 and 54.2) on the defined 129S6 genetic background.

### Other surfactant components modify the outcome of RSV infection

Additional protein and lipid components of pulmonary surfactant have been reported to influence the pulmonary response to RSV infection. Previous studies have shown that SP-A and SP-D also reduce injury from RSV infection. Both SP-A and SP-D (*Sftpa-/-, Sftpd -/-*) deficient mice were more susceptible to RSV infection. Co-administration of isolated SP-A with virus at the time of instillation reduced the cellular inflammation and increased viral clearance from the lungs of *Sftpa -/-* mice [28,29]. However neither SP-A nor D levels were altered in the BALF from lungs of RSV infected *Sftpc -/-* mice relative to *Sftpc +/+* mice [14]. Thus neither SP-A nor SP-D influenced RSV infection in the SP-C deficient lung.

In the present study, vesicles generated with the major representative surfactant phospholipid species (DPPC and POPC) did not reduce the viral infection *in vitro*. The DPPC:POPC vesicles complexed with SP-C decreased RSV infectivity indicating that SP-C associated with major surfactant phospholipids confers a protective role during RSV infection. The phospatidylglycerol (PG) derivative palmitoyloleoyl-phosphatidylglycerol (POPG) is found in pulmonary surfactant and was shown to independently suppress RSV infectivity [30]. The activity of pure POPG was noted at concentrations eight fold above phospholipid levels tested in this study. It is unknown if POPG in surfactant-like phospholipid mixtures would confer antiviral activity on the order of our SP-C dependent findings. While not directly comparable, the POPG activity could exert a synergistic affect with SP-C to modify the outcome of RSV infection. The surfactant phospholipid composition of *Sftpc -/-* mice was unchanged from that of *Sftpc +/+* mice inferring that there is no change in potential PG derived phospholipid antiviral activity between the two *Sftpc* genotypes [8].

### Immuno-protection by SP-C extends to other lung pathogens

The function of SP-C as a key protective component of surfactant was identified in a model of influenza A infection. The commercial surfactant extract Surfacten® acted as an adjuvant increasing the effective nasal mucosal immunity upon viral challenge [31]. Surfacten lacks SP-A and SP-D and is enriched in SP-C. Representative surfactant phospholipid and protein mixtures were assessed to identify active components that confer the viral immune response. Synthetic phospholipid mixtures similar to the commercial Surfacten preparation alone or in combination with SP-B did not induce a protective antiviral response. However addition of SP-C to the phospholipid preparations produced a nasal IgA response equivalent to the Surfacten-influenza mixture [32]. These findings on SP-C mediated influenza protection along with the current SP-C regulation of RSV injury directly infer an immune-protective role for SP-C independent of representative surfactant phospholipids alone.

Some *SFTPC* patient histories document recurrent infections. These findings are supported by a bacterial infection study in the *Sftpc -/-* mice demonstrating reduced clearance of the respiratory pathogen *Pseudomonas aeruginosa*[33]. Taken together these reports identify a protective role for SP-C during both bacterial and viral infections.

### The Toll-like receptor TLR3 influences the type II cell response in SP-C deficient mice

While the epithelial response to RSV is complex, cytokine activation and the activity of the Toll-like innate immune receptors have been shown to be integral to the inflammatory and epithelial protective reaction to RSV [34,35]. Because SP-C is synthesized and secreted in the alveolus we hypothesized that the increased susceptibility to infection resulted from compromised type II cells. *Sftpc -/-* type II cells had increased basal levels of key cytokines and expressed increased cytokine levels when challenged *in vitro* with either the TLR3 specific ligand poly I:C, or RSV infection. TLR3 has previously been shown to mediate immune response to RSV infection and SP-C blocked TLR3 signaling *in vitro*[14,36]. In addition *Sftpc -/-* mice had increased lung inflammation when challenged *in vivo* with poly I:C. Our findings indicate that the absence of SP-C is sufficient to predispose the type II cell to viral infection and stimulate TLR3 mediated inflammatory responses. TLR3 is associated with intracellular signaling vesicles and SP-C is an obligate membrane or phospholipid associated lipopeptide. The increased intrinsic expression of inflammatory cytokines by SP-C deficient type II cells suggest the interpretation that under normal conditions there is intracellular vesicle-to-vesicle interaction where SP-C restrains unwarranted or non-specific activation of TLR3 inflammatory signaling. Collectively our results support the concept that type II cells contribute to alveolar defense during severe RSV infection that in part relies upon the immune-protective activity of SP-C.

## Conclusions

RSV and other viral infections exacerbate lung disease in young individuals with abnormalities of SP-C. A genetic model of regulated SP-C restoration was generated and used to determine efficacy of SP-C replacement to reduce RSV exacerbations in the lungs of SP-C deficient mice. RSV induced pulmonary inflammation was decreased by induced SP-C replacement prior to infection. These findings indicate that available commercial surfactant extracts containing SP-C may have therapeutic value to individuals known to have impaired SP-C production. Type II cells isolated from the lungs of *Sftpc -/-* mice and maintained in culture produced an increased inflammatory response to RSV indicating that the susceptibility to RSV exacerbation occurs in part from an overt response by the alveolar epithelium. Our findings infer that SP-C abnormalities can distort the alveolar innate immune response.

## Competing interests

The authors have no competing interests to declare.

## Authors’ contributions

SWG and TRK designed, supervised experiments, data analysis, prepared and revised manuscript and prepared figures (SWG). APS prepared virus and viral infections and revised manuscript. MDM and TLR conducted cell transfections, type II cell isolations, *in vivo* infection experiments and prepared figures. JEB prepared SP-C reagents for viral inhibition studies prepared and revised manuscript. KP completed cytokine expression studies of isolated type II cells and revised manuscript. All authors read and approved the final manuscript.
